# The Influence of Chromosomal Polymorphism on Embryo Development and Embryonic Molecular Karyotype in Preimplantation Genetic Testing for Chromosomal Translocation

**DOI:** 10.3389/fphys.2020.543188

**Published:** 2020-11-26

**Authors:** Gang Li, Weiyi Shi, Wenbin Niu, Jiawei Xu, Yihong Guo, Yingchun Su, Yingpu Sun

**Affiliations:** Reproductive Medical Center, Henan Province Key Laboratory for Reproduction and Genetics, The First Affiliated Hospital of Zhengzhou University, Zhengzhou, China

**Keywords:** next-generation sequencing, embryo development, aneuploid, preimplantation genetic testing, chromosomal polymorphism

## Abstract

Traditionally, chromosomal polymorphisms (CPMs) are normal genetic variants in individuals with no phenotypic variations. However, some studies have shown that CPM is related to reproductive diseases. We explored the influence of CPM on embryonic development and molecular karyotype in chromosomal translocation (CT) patients undergoing preimplantation genetic testing (PGT) between February 2013 and May 2019. Twenty-six cases with CPM and 56 controls with normal chromosomes were included. Furthermore, a 1:4 match pair analysis by female age included 39 cases with CTCPM and 185 controls with CT. There was no statistical difference in fertilization rate (78.48% vs. 78.33%), cleavage rate on Day 3 (90.32% vs. 89.16%), blastocyst rate (60.00% vs. 60.80%), and the high-quality blastocyst rate (36.31% vs. 35.22%) between CPM and normal chromosomes. The high-quality blastocyst rate of CTCPM was significantly lower than that for CT (26.78% vs. 38.89%). Moreover, there was no statistical difference in fertilization rate (70.65% vs. 70.37%), cleavage rate on Day 3 (88.67% vs. 89.53%), and blastocyst rate (48.48% vs. 53.17%) between CTCPM and CT. In addition, one CTCPM spouse had a lower high-quality blastocyst rate, especially of males with CTCPM. Abnormal embryo rates of CTCPM were significantly higher than those for CT (78.64% vs. 68.93%). Abnormal embryo rates were higher in both CTCPM and CPM paternal carriers with CT partners, respectively. For CT, CTCPM may have an impact on the high-quality blastocyst rate and embryonic molecular karyotype, especially in male patients. Patients with CTCPM are relatively rare, but this population would benefit from being explored using a larger sample size.

## Introduction

Chromosomal polymorphism (CPM) is known as some minor variation of banding karyotype in normal people, and it is constant in an individual. Most variations consist of heterochromatic regions, which are enriched with highly repetitive satellite DNA sequences. These are located in the non-coding sequences of DNA and often occur in the long or short arms of chromosomes 1, 9, and 16. Polymorphisms of D and G group chromosomes (13, 14, 15, 21, and 22) are included in the short arms, the satellites, and stalks of acrocentric chromosomes. Pericentric inversions on chromosomes 1, 2, and 9, and pericentric inversions and the size of long or short arms of chromosome Y are also included.

Traditionally, CPM is a normal variant in an individual with no phenotypic variation. However, some studies have shown that CPM may be related to cancer (Pei and Zhang, 2013; Rojas et al., 2014; Zhi et al., 2017) and reproductive diseases. Madon and Athalye (2005) showed that CPM accounts for a high proportion of patients with primary infertility or repeated miscarriages. Some studies show that CPMs are independently associated with multinucleated embryo formation (Sun et al., 2018). It is even suggested that male CPMs are linked to a higher cumulative early miscarriage rate and lower live birth rate following *in vitro* fertilization (IVF) (Ni et al., 2017). However, some studies show that CPM has no effect on IVF/intracytoplasmic sperm injection (ICSI) outcome (Ou et al., 2019).

In the current study on chromosomal translocation (CT) carriers, clinical and laboratory data of preimplantation genetic testing (PGT) were analyzed for CTCPM carriers and CT carriers, in order to explore the influence of CPM on embryo development and embryonic molecular karyotype in CT carriers.

## Materials and Methods

### Participants

We retrospectively analyzed the clinical and laboratory data of PGT for CPMs, CTCPMs, CTs, and normal chromosomes at the Reproductive Medicine Center of the First Affiliated Hospital of Zhengzhou University from February 2013 to May 2019. All study methods were approved by the Institutional Review Board and Ethics Committee of the First Affiliated Hospital of Zhengzhou University and were conducted in accordance with relevant guidelines and regulations. All subjects enrolled in the study gave written formal consent for their participation.

The chromosome karyotype analysis was assessed in peripheral blood lymphocytes of parents. Peripheral blood samples 3 to 5 mL were extracted; 0.5 mL was inoculated into peripheral blood lymphocyte culture medium, mixed upside down, and placed in an incubator without CO_2_ at 37°C for culture. After the above cells were cultured for 3 days, 60 μg/mL of colchicine with a concentration of 20 μg/mL was added. Colchicine was transferred to a 37°C constant temperature incubator for further culture for 1 h, and then conventional cell harvesting was performed, prefixation by hypo-osmosis, dropping tablet at 56°C, and baking tablet at 65°C, and G banding was used to capture the karyotype by automatic chromosome scanning system GSL-120 (Leica, Germany). At least five karyotypes were analyzed in each case, and 20 metaphase fission phases were counted. Twenty-six cases with CPM (30 cycles) and 56 controls with normal chromosomes (66 cycles) were investigated. Because the CTCPM carriers were far fewer than the CT carriers, a 1:4 match pair analysis ultimately identified 39 cases with CTCPM (47 cycles) and 185 controls with CT (188 cycles). Pairs were matched based on female age. The CTCPM carriers were divided into two subgroups: one spouse with CPM and a partner with CT, or one spouse with CTCPM and a partner with no abnormalities.

### PGT Procedure

One female underwent an early follicular phase long-acting regimen for controlled ovarian stimulation (Kong et al., 2018), and six females underwent a modified super-long protocol (half dose of long-effect gonadotropin-releasing hormone-a was injected at the same time as a short-acting long luteal phase stimulation protocol, whereas the other half was given a second injection on the 28th day after the first injection). After reaching the standard for down-regulation, human chorionic gonadotropin (hCG) (Ovidrel; Merck Sereno), either alone or combined with the hCG 2,000 IU, was injected as a trigger. The remainder of the females underwent short-acting long luteal phase stimulation protocols (Jin et al., 2019). Oocytes were observed closely via transvaginal ultrasound. When the diameters of more than two dominant follicles were greater than 18 mm, hCG was used. 38 h later, the oocytes were retrieved under the guidance of transvaginal ultrasonography.

Mature oocytes (MII) obtained after oocytes retrieval were fertilized by ICSI. Meanwhile, the obtained sperm by masturbation was enriched by gradient centrifugation. And then, it was added into the ICSI dish, and an alive and well-formed sperm was selected and pressed vertically at the lower part of the sperm tail to immobilize. The sperm was absorbed by the injection needle, and the sperm was injected into the ooplasm after the oocyte was fixed. After ICSI, either G1-Plus (Vitrolife, Sweden) or K-SICM (Cook, Australia) microdroplets were used in the embryo culture medium, and the embryos were transferred to G2-Plus (Vitrolife, Sweden) or K-SIBM (Cook, Australia) microdroplets on the 3rd day. All embryos were cultured at 37°C in a 6% CO_2_ and 5% O_2_ incubator.

On the 3rd day of operation, the zona pellucida of embryo was drilled with the laser and then continued to be cultured in the blastocyst culture incubator. The blastocyst formed on the 5th or 6th day could be seen that trophoblast ectoderm cells were hatched from the pores. Two to six trophoblast ectoderm cells were absorbed by biopsy needle in the biopsy dish and placed at 37°C in an aerated incubator for PGT.

Subsequently, single-nucleotide polymorphism (SNP) microarray or multiple annealing and looping-based amplification cycle (MALBAC) next-generation sequencing technology were used to assess these biopsied cells. Whole-genome amplification for trophectoderm (TE) cells obtained via biopsy was conducted using the QIAGEN REPLI-g Single Cell kit. After cell lysis, the samples were incubated in the polymerase chain reaction (PCR) machine and then placed at room temperature. The amplifications were microarray by HumanCytoSNP-12 microarray (Illumina) for SNP microarrays. On another way, the samples were preamplified in the PCR machine in the MALBAC preamplification mixture and then placed at room temperature and then incubated in the PCR machine in the MALBAC amplification mixture. The MALBAC amplification products were purified, and the data were sequenced by Illumina Hiseq 2500 using an ultrahigh-throughput sequencing system. After biopsied cell collection, the blastocyst was quickly transferred to a four-hole plate and put into an incubator at 37°C and 6% CO_2_. The blastocysts were evaluated on Day 5/6 according to Gardener score: those scoring ≥ 3 BB were considered high-quality blastocysts. After embryo biopsy, vitrification was performed, and embryos identified as balanced or normal underwent frozen embryo transfer.

### Statistical Methods

Baseline materials were analyzed by independent *t* testing. The rates were compared by χ^2^ test and Fisher exact probabilities, and *P* < 0.05 was considered statistically significant. The test level of pairwise comparisons among three groups was corrected by Bonferroni tests, and *P* < 0.02 was considered statistically significant.

## Results

### Baseline Materials

All patients recruited were diagnosed by karyotype analysis of peripheral blood. First, the baseline data of 26 cases with CPM, including 30 cycles, and 56 controls with normal chromosomes, including 66 cycles, were not significantly different. Thirty-nine couples including CTCPM carriers and 186 CT carriers performed 47 and 188 PGT cycles, respectively. With the exception of body mass index (BMI), there were no significant differences between the two groups within the baseline information (Table 1).

**TABLE 1:** General information for patients and result of PGT.

	**Chromosomal polymorphism**	**Normal chromosomes**	***P***	**Chromosomal polymorphism combined with translocation**	**Chromosomal translocation**	***P***
Female age	34.27 ± 6.28	35.09 ± 5.47	0.52	28.97 ± 3.53	29.45 ± 3.42	0.68
Male age	35.73 ± 7.00	36.36 ± 6.31	0.66	30.18 ± 4.23	30.14 ± 4.11	0.97
Female BMI	23.72 ± 2.12	22.81 ± 2.89	0.13	21.55 ± 2.72	22.93 ± 2.89	0.01
Female AFC	14.43 ± 5.22	13.48 ± 6.57	0.49	15.10 ± 7.36	16.13 ± 5.80	0.49
No. of retrieved oocytes	15.57 ± 11.25	13.79 ± 7.63	NS	16.74 ± 9.88	16.66 ± 8.53	NS
MII oocytes rate (%)	84.58% (395/467)	86.7% (789/910)	0.28	88.31% (695/787)	87.96% (2,755/3,132)	0.79
Fertilization rate (%)	78.48% (310/395)	78.33% (618/789)	0.95	70.65% (556/787)	70.37% (2,204/3,132)	0.88
No. of biopsied blastocysts	149	293	NS	215	944	NS
No. of aneuploidy embryos	86	204	NS	162	610	NS
Aneuploidy rate	61.87% (86/139)	70.34% (204/290)		78.64% (162/206)	68.93% (610/885)	0.01

### The Distribution of CPM

CPM in humans is usually located in secondary constrictions, centromeres, satellites, and the distal long arms of Y chromosomes. Variants of the long arm of chromosome 1, 9, and 16 included 11 cases in couples with CTCPM and 5 cases in patients with CPM. Polymorphisms of D and G group chromosomes (13, 14, 15, 21, and 22) included 5 cases in couples with CTCPM and 6 cases in patients with CPM. There were 16 cases with pericentric inversions on chromosomes 1 and 2, and 9 in couples with CTCPM, and 7 cases in patients with CPM. In addition, eight cases in couples with CTCPM and six cases in patients with CPM were recruited with polymorphism of chromosome Y.

### PGT Results

#### Embryo Development Outcomes

In patients with CPM, 467 oocytes were collected, 395 of them were mature metaphase II stage (MII), and 310 (78.48%) fertilized normally. After fertilization, 280 embryos (90.32%) developed to Day 3, and 168 (60.00%) embryos developed to blastocysts on Day 6, of which 61 (36.31%) were evaluated as high-quality blastocysts. For patients with normal chromosomes, 910 oocytes were retrieved; 789 were MII, and 618 (78.33%) were fertilized normally. The number of embryos on Day 3 was 551 (89.16%), and 335 (60.80%) of them reached blastocyst stage, of which 118 (35.22%) were scored as high-quality blastocysts. When comparing the two groups, there were no statistical differences in fertilization rate, cleavage rate on Day 3, blastocyst rate, and high-quality blastocyst rate between the two groups (*P* = 0.95, *P* = 0.59, *P* = 0.82, and *P* = 0.81, respectively; Table 1).

In patients with CTCPM, a total of 787 oocytes were retrieved, 695 of them were mature MII, and 556 (70.65%) fertilized normally. After fertilization, 493 (88.67%) embryos developed to Day 3, and 239 (48.48%) embryos developed to blastocysts on Day 6, of which 64 (26.78%) were evaluated as high-quality blastocysts. For patients with CT, 3,132 oocytes were collected; 2,755 were MII, and 2,204 (70.37%) were fertilized normally. The number of embryos on Day 3 was 1,973 (89.53%), and 1,049 (53.17%) of them reached blastocyst stage, of which 408 (38.89%) were scored as high-quality blastocysts. However, when the two groups were compared, the high-quality blastocyst rate of patients with CTCPM was significantly lower (χ^2^ = 12.31, *P* = 0.00). Furthermore, there was no statistical difference in fertilization rate, cleavage rate on Day 3, and blastocyst rate between the two groups (*P* = 0.88, *P* = 0.56, *P* = 0.06; Table 1).

Further analysis showed that one spouse with CTCPM had a lower high-quality blastocyst rate (χ^2^ = 10.42, *P* = 0.00), especially for male embryos (χ^2^ = 15.25, *P* = 0.00; Table 2).

**TABLE 2:** The result of embryos development.

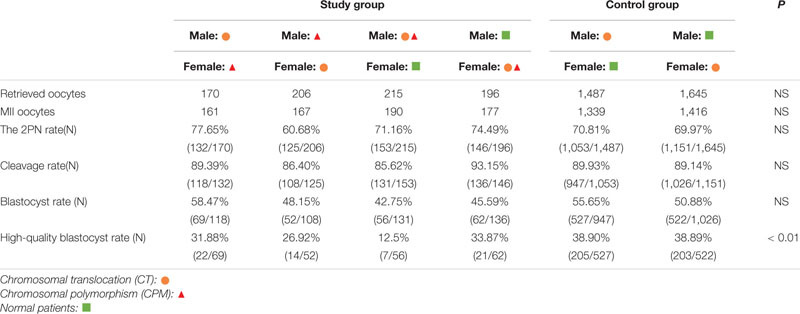

#### Outcomes of Embryonic Molecular Karyotype

**FIGURE 1:**
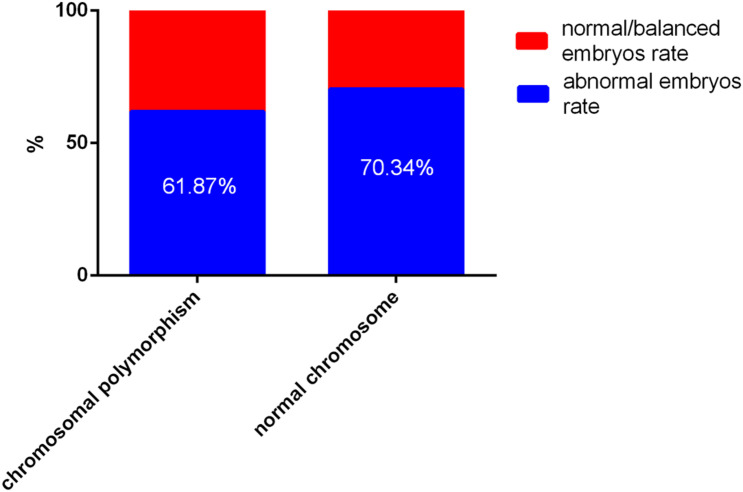
The outcome of embryos after preimplantation genetic testing (PGT) for chromosomal polymorphism and normal chromosomes Of a total of 427 embryos, 8 failed to be amplified. For patients with chromosomal polymorphism, 53 (38.13%) of 139 blastocysts were normal or balanced, and 86 (61.87%) were abnormal. For patients with normal chromosomes, 275 (31.07%) of 290 were normal or balanced, and 204 (70.34%) had an abnormal molecular karyotype.

According to blastocyst grading, the numbers of blastocysts biopsied in patients with CPM and patients with normal chromosomes were 145 and 292, respectively. For patients with CPM, 6 were not diagnosed successfully, 53 (38.13%) blastocysts were normal or balanced, and 86 (61.87%) had an abnormal molecular karyotype. For patients with normal chromosomes, 2 were amplified abortively, 86 (29.66%) blastocysts were normal or balanced, and 204 (70.34%) had an abnormal molecular karyotype. When comparing the two groups, there was no statistical difference in abnormal embryos rates (Figure 1).

**FIGURE 2:**
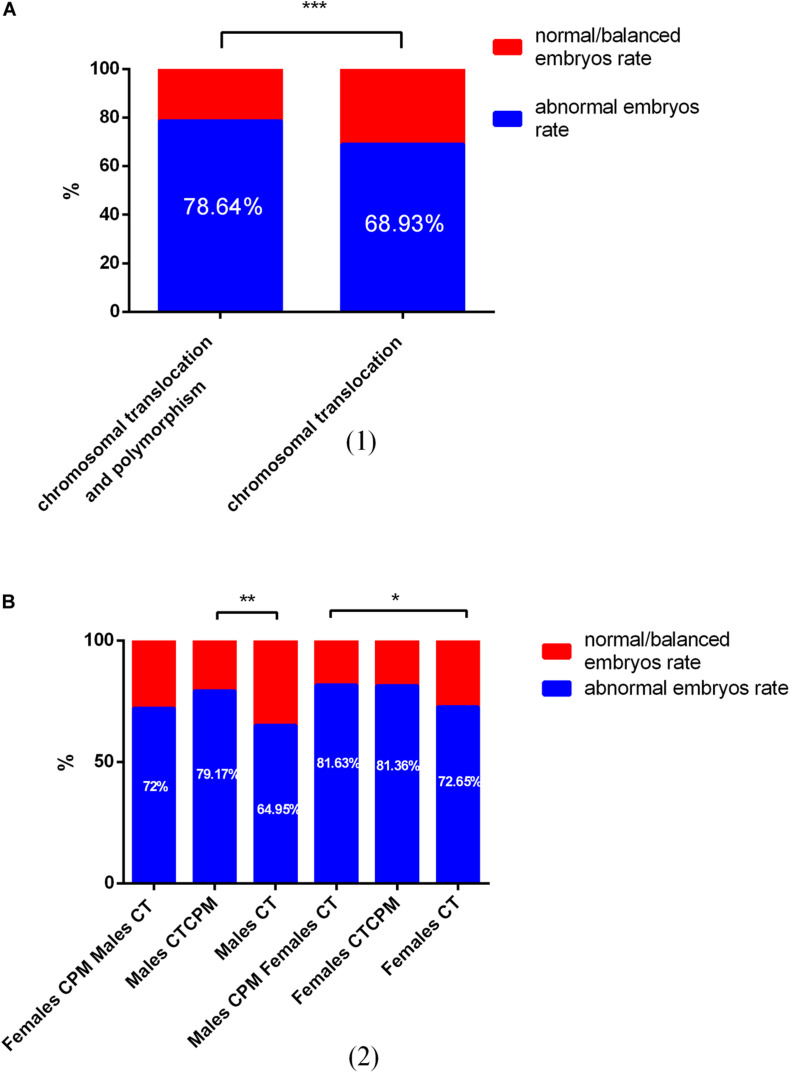
The outcome of embryos after preimplantation genetic testing (PGT) for chromosomal translocation (CT) and polymorphism and CT **(A)** Of a total of 1,172 embryos, 79 failed to be amplified. For patients with CT and polymorphism, 44 (21.34%) of 206 blastocysts were normal or balanced, and 162 (78.64%) were abnormal. For patients with CT, 275 (31.07%) of 885 were normal or balanced, and 610 (68.93%) had an abnormal molecular karyotype. **(B)** For males with chromosomal polymorphism (CPM) and females with CT, normal or balanced blastocysts vs. abnormal were 72% vs. 28%. For males with CPM combined with translocation (CTCPM), normal or balanced blastocysts vs. abnormal were 79.17% vs. 20.83%. For males with CT, normal or balanced blastocysts vs. abnormal were 64.95% vs. 35.05%. For females with CPM and males with CT, normal or balanced blastocysts vs. abnormal were 81.63% vs. 18.37%. For females with CTCPM, normal or balanced blastocysts vs. abnormal were 81.36% vs. 18.64%. For females with CT, normal or balanced blastocysts vs. abnormal were 72.65% vs. 27.35%.

The numbers of blastocysts biopsied in patients with CTCPM and patients with CT were 215 and 957, respectively. For patients with CTCPM, 9 were not diagnosed successfully, 44 blastocysts (21.34%) were normal or balanced, and 162 (78.64%) had an abnormal molecular karyotype. For patients with CT, 72 of a total of 957 were amplified abortively, 275 (31.07%) blastocysts were normal or balanced, and 610 (68.93%) had an abnormal molecular karyotype. When comparing the two groups, abnormal embryo rates were significantly higher in patients with CTCPM (χ^2^ = 7.62 *P* = 0.01; Figure 2).

Among these cases, when compared with males with CT, abnormal embryos rates were higher in both paternal CTCPM carriers and paternal CPM carriers with partners carrying CT, respectively (χ^2^ = 15.25, *P* = 0.00; χ^2^ = 5.50, *P* = 0.02). However, when compared with females with CT, there were no significant differences in maternal carriers (χ^2^ = 0.59, *P* = 0.44; χ^2^ = 0.25, *P* = 0.62; Table 3).

**TABLE 3:** The outcomes of embryonic molecular karyotype.

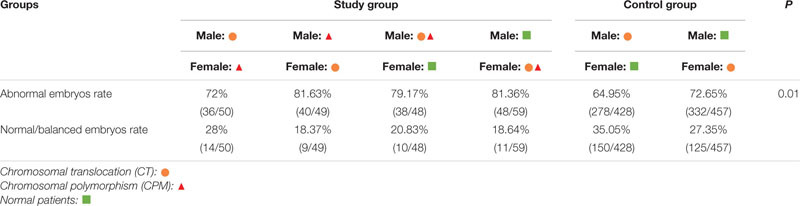

## Discussion

CPM is regarded as a normal variation in individuals, but recent studies have shown that CPM has a notable impact on reproductive outcome. A meta-analysis showed that during IVF or ICSI, male partners with CPM had negative effects on fertilization rate, embryo cleavage rate, high-quality embryo rate, and live birth rate, but there were no significant differences in early spontaneous abortion rate, clinical pregnancy rate, or ongoing pregnancy rate (Ou et al., 2019). A multicenter medical study in Brazil showed that CPM accounts for an important proportion of both male and female carriers in couples who have had a first or second miscarriage (Cavalcante et al., 2020). In males with oligozoospermia, the detection rate of CPM was also increased (Chen et al., 2017; Dai et al., 2018), and it showed that polymorphic variations on the Y chromosome were the most prevalent polymorphism in infertile men, especially in patients with severe oligozoospermia (Guo et al., 2012). Thus, CPM does not appear to be the absence of an individual phenotype.

Because of the known inverse relationship between advanced maternal age (>35 years) and embryo euploidy (Demko et al., 2016), pairs were matched based on female age in order to minimize the influence of female age on embryo molecular karyotype results. Although BMI figures between the two groups were statistically different, there was no statistically significant relationship between BMI and euploidy (Goldman et al., 2015). We tried to explore the influence of CPM on embryo development and embryonic molecular karyotype in CT carriers. First, the CPM carriers were compared with patients with normal chromosomes. Then, the PGT data were analyzed for CTCPM carriers and CT carriers.

Embryo development suggests that CPM is associated with multinucleated embryo formation (Sun et al., 2018) and has an impact on fertilization rate, embryo cleavage rate, high-quality embryo rate, and live birth rate (Liang et al., 2014; Ou et al., 2019). However, in our data, there was no statistical difference in fertilization rate, cleavage rate on Day 3, blastocysts rate, or high-quality blastocyst rate between CPM carriers and patients with normal chromosomes. There was also no significant difference in fertilization rate, cleavage rate on Day 3, and blastocyst rate in patients with CTCPM, which was not similar to the previously mentioned study. However, it indicated that ICSI may increase the fertilization rate for men with CPM (Liang et al., 2014). Thus, the fertilization rate of our study showed no significant difference. The high-quality blastocyst rate decreased, and the differences were mainly observed when one spouse had CTCPM, especially male patients, which was consistent with other studies. The study showed that variability in the retention of nucleosomes could interfere with constitutive heterochromatin function in the zygote and so affect the developmental potential of the embryo (Ou et al., 2019). These results may suggest that for CT, CPM may not affect embryo development, but it can decrease the high-quality blastocyst rate in ICSI-PGT.

Our data showed that although the abnormal embryo rates of CPM were higher than normal, there was no statistical difference. However, compared with the CT group, abnormal embryos rates were significantly higher in patients with CTCPM, which was mainly attributed to one spouse having CTCPM, particularly the male partner. This may indicate that in patients with CT, CPM amplified its effects, rather than remaining inactive. In colorectal cancer, chromosomal instability is also proposed to shape aneuploidy landscapes (Bolhaqueiro et al., 2019). Morales et al. show that CPM could affect meiotic segregation in male gametes and lead to a higher rate of aneuploid embryos (Morales et al., 2016), which was similar to our findings. Another study suggests that segmental aneuploidy could affect paternal chromosomes compared with whole chromosomal aneuploidy in human IVF embryos (Kubicek et al., 2019). Furthermore, it may be more difficult for males with CTCPM to obtain a normal or balanced embryo via PGT. Heterochromatin located in the centromere has an important role in spindle attachment and chromosome movement; thus, chromosome pairing or sister chromatid cohesion may be disturbed owing to its alteration during mitosis and meiosis (Guo et al., 2012; Kubicek et al., 2019), which in turn results in embryo aneuploidy.

There were some limitations to our study. Because of the relative rarity of patients with CTCPM, the sample size of the study group was small; in future studies, it would be important to expand the sample size.

## Data Availability Statement

The genotype and sequencing data has been deposited into Figshare (link: https://figshare.com/articles/dataset/For_Frontiers_in_Physiology/13128011/1).

## Ethics Statement

The studies involving human participants were reviewed and approved by Institutional Review Board and Ethics Committee of the First Affiliated Hospital of Zhengzhou University. The patients/participants provided their written informed consent to participate in this study. Written informed consent was obtained from the individual(s) for the publication of any potentially identifiable images or data included in this article.

## Author Contributions

GL conceived and designed the experiments. YG and YCS selected and supervised suitable patients. GL performed comprehensive chromosome screening. WN and JX performed next-generation sequencing and sequencing data analysis. YCS and YG recruited the patients, retrieved oocytes, and transferred embryos. YPS provided overall supervision. GL and WS drafted the manuscript. All authors reviewed the final manuscript.

## Conflict of Interest

The authors declare that the research was conducted in the absence of any commercial or financial relationships that could be construed as a potential conflict of interest.
